# A semantic analysis-driven customer requirements mining method for product conceptual design

**DOI:** 10.1038/s41598-022-14396-3

**Published:** 2022-06-16

**Authors:** Xuan-Yu Wu, Zhao-Xi Hong, Yi-Xiong Feng, Ming-Dong Li, Shan-He Lou, Jian-Rong Tan

**Affiliations:** 1 State Key Laboratory of Fluid Power and Mechatronic Systems, Zhejiang University, Hangzhou, 310027 China; 2 Engineering Research Center for Design Engineering and Digital Twin of Zhejiang Province, Zhejiang University, Hangzhou, 310027 China

**Keywords:** Mechanical engineering, Information technology

## Abstract

Precise customer requirements acquisition is the primary stage of product conceptual design, which plays a decisive role in product quality and innovation. However, existing customer requirements mining approaches pay attention to the offline or online customer comment feedback and there has been little quantitative analysis of customer requirements in the analogical reasoning environment. Latent and innovative customer requirements can be expressed by analogical inspiration distinctly. In response, this paper proposes a semantic analysis-driven customer requirements mining method for product conceptual design based on deep transfer learning and improved latent Dirichlet allocation (ILDA). Initially, an analogy-inspired verbal protocol analysis experiment is implemented to obtain detailed customer requirements descriptions of elevator. Then, full connection layers and a softmax layer are added to the output-end of Chinese bidirectional encoder representations from Transformers (BERT) pre-training language model. The above deep transfer model is utilized to realize the customer requirements classification among functional domain, behavioral domain and structural domain in the customer requirement descriptions of elevator by fine-tuning training. Moreover, the ILDA is adopted to mine the functional customer requirements that can represent customer intention maximally. Finally, an effective accuracy of customer requirements classification is acquired by using the BERT deep transfer model. Meanwhile, five kinds of customer requirements of elevator and corresponding keywords as well as their weight coefficients in the topic-word distribution are extracted. This work can provide a novel research perspective on customer requirements mining for product conceptual design through natural language processing.

## Introduction

Product conceptual design plays an important role in the product lifecycle, which determines product’s primary cost with a small investment^1^. Defects caused by insufficient product conceptual design are difficult to be remedied in the manufacturing and maintenance stages. This stage starts from the customer requirements analysis, then gradually realizes the mapping from product functional to physical structure, and obtains the design scheme through evaluation and optimization in final^2^. Customer-centered product design philosophy is widely recognized by manufacturing enterprises nowadays. Therefore, narrowing the gap between product design and customer requirements is a pivotal goal from beginning to end. Previous published studies conduct customer investigations by questionnaire or interview to gather data for analyzing customer requirements. These offline methods are complicated and time-consuming. For the past few years, a large quantity of literature has researched the extraction of customer requirements from online comments^3,4^. However, traditional studies failed to consider customer requirements representation in the analogical reasoning environment so that it is not effortless to gain latent and innovative customer requirements. Some studies have indicated that accommodating customers into the analogical reasoning environment is essential^5,6^. In addition to explicit customer requirements, latent customer requirements are extremely crucial to product innovation and success. Understanding and acquisition of latent customer requirements have prominent effect on satisfying customers. While explicit customer requirements tend to be self-evident as long as we can obtain raw data like questionnaire and interview, latent customer requirements are usually hidden in the semantics of customer requirements information and customers may not be distinctly aware of them. Analogical reasoning in the product conceptual design is the process of solving current design problems based on the solutions of past design problems^5^. Design process can be supported using analogical stimuli by assisting participants to overcome fixation and generate abundant solutions with more positive characteristics during ideation. In the customer requirements analysis stage, customers have established a preliminary perceptual cognition when they interact with product function and structure. If customers are placed in the analogical reasoning environment, the product feedback information is processed ulteriorly in their brain according to the given analogical stimuli, invisible and creative product requirements existing in the customer’s brain can be discovered. Meanwhile, it is inevitable that analogical stimuli results are not always positive and can be incorrect in the far-domain stimuli environment especially. In order to ensure a maximal utility for analogical stimuli, near-domain stimuli are provided to guarantee the feasibility and usefulness of the customer requirements and far-domain stimuli are selected to assure the novelty of the customer requirements. Besides, the collected customer requirements should be carefully evaluated and filtered by the domain experts.

VPA is an effective research paradigm that can reflect the cognitive process by thinking aloud^7,8^. The subjects elaborate their detailed thinking content when performing a specific cognitive task truthfully. The obtained recordings are converted into texts so that the thinking process of subjects can be displayed explicitly. Besides, an analogy-inspired VPA experiment is a practical engineering method, which acquires inspiration for current cognition task by utilizing the solution space of other domains^9,10^. In this paper, the subjects express the functional, behavioral and structural customer requirements of elevator based on the existing design scheme and the visual stimuli including near and far domain. The specific definitions of function, behavior and structure are demonstrated here^11^. Firstly, the function represents the abstract description about product’s working capability. Then, the behavior indicates the scientific principle of realizing the function. Lastly, structure refers to the physical entity that produces the desired behavior and performs the predetermined function. Designers always follow the function-behavior-structure design process model, which helps designers to solve specific design tasks by describing the relationship among function, behavior and structure^11^. The reason why customer requirements are classified into the above three categories is that it is beneficial to express customer requirements in accordance with the cognition of designers. Hence, this can not only discover the potential product internal requirements involving function, behavior, structure as well as associated constraints, but also accelerate the subsequent solving process of product design schemes for designers. There are plentiful common features between near-domain stimuli and design problem, and they all come from the same or similar fields. It is easier for subjects to propose feasible customer requirements. On the contrary, there are few common features between far-domain stimuli and design problem, which contributes to the generation of innovative customer requirements^9^. Hence, the text data transformed from VPA data is segmented with natural sentences as the unit and then input into the established BERT deep transfer model. The functional, behavioral and structural customer requirements are classified by fine-tuning the BERT deep transfer model. In fact, a large amount of requirement texts is hard to provide direct assistance to designer after the customer requirements classification. Thus, acquiring the practicable customer requirements is actually a topic analysis task. Functional customer requirements representing customer intention maximally are chosen as the main data source of customer requirements mining. Topic modeling is carried out for the functional requirement semantics. Aiming at existing problems such as the frequency representation of document-word matrix and the empirical selection of topic quantity about traditional latent Dirichlet allocation, ILDA is proposed to enhance the application efficacy in the customer requirements mining.

To overcome the limitations of prior approaches, this paper proposed a semantic analysis-driven customer requirements mining method for product conceptual design based on deep transfer learning and ILDA. In comparison with the existing studies, our work makes three distinctive contributions: (1) An analogy-inspired VPA experiment providing near-domain and far-domain stimuli is presented for obtaining feasible and innovative customer requirement descriptions of elevator. Latent customer requirements are usually hidden in the semantics of customer requirements information and customers may not be distinctly aware of them. Traditional studies failed to consider customer requirements representation in the analogical reasoning environment so that it is not effortless to gain potential and innovative customer requirements. (2) A BERT deep transfer model is established to realize the customer requirements classification among functional domain, behavioral domain and structural domain by fine-tuning training. It can be seen from the final results that satisfying overall classification accuracy and well-pleasing efficacy for Precision, Recall and F1 can be obtained. (3) The ILDA is proposed to mine the functional customer requirements representing customer intention maximally. The optimal topic quantity is determined based on the proposed measurable indicator Perplexity-AverKL. The implicit customer requirements of elevator can be elaborated by topic-word distribution explicitly. In general, this work can provide a novel research perspective on customer requirements mining for product conceptual design by analogy-inspired VPA experiment and natural language processing algorithms.

**Figure 1:**
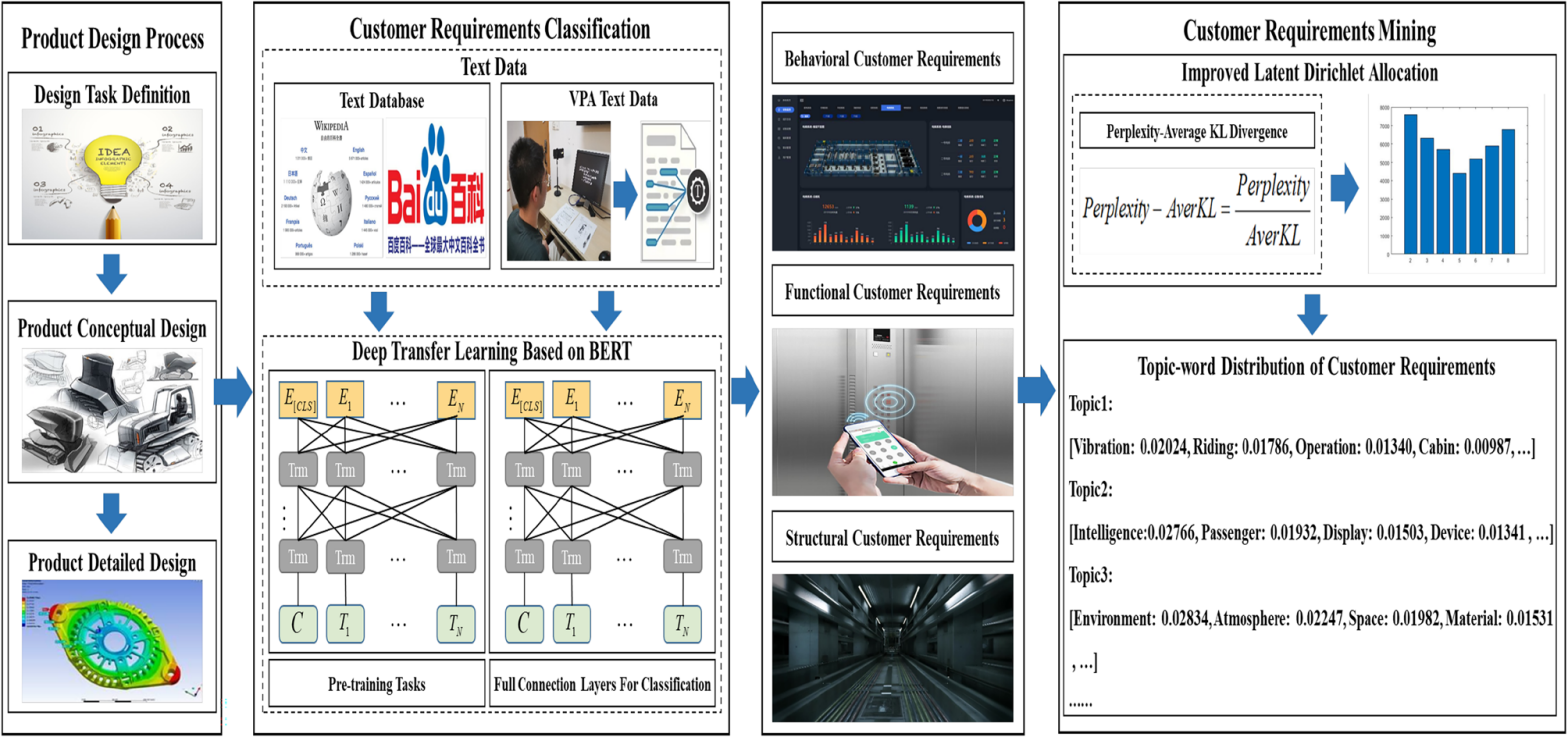
The overall implementation framework.

The overall implementation framework is displayed in Fig. 1. The rest of this paper is organized as follows. “Literature review” section reviews the related work. “Method” section illustrates the customer requirements classification based on BERT and customer requirements mining based on ILDA. A case study is presented in “Case study” section. Finally, “Conclusions” section concludes our work.

## Literature review

This Section is closely divided into three parts. The one subsection describes the research situation of customer requirements classification, and another subsection introduces the deep transfer learning in the natural language processing, and a third subsection elaborates the customer requirements mining.

### Customer requirements classification

Due to the diversity, dynamics and fuzziness of customer requirement semantics, it is inevitable to classify them systematically in order to understand and further analyze them. Scholars at home and abroad have studied it from different perspectives. Kano model as well as its derivatives is an available requirements analysis tool, which distinguishes the different nonlinear relationships between customer requirements fulfillment and customer satisfaction^12^. Xu et al.^13^ presented an analytical Kano model to classify functional requirements into logical groups. This leads to an optimal trade-off between customer classification and producer capability. Lou et al.^14^ proposed a data-driven approach for customer requirements discernment via Kano model, intuitionistic fuzzy sets theory and electroencephalogram technology. The vagueness of requirements is handled at the semantic expression and neurocognitive level. Shi et al.^15^ utilized big data of online customer reviews and improved Kano model to classify customer requirements accurately and efficiently. Polynomial modeling and least square methods are adopted to define customer satisfaction and function implementation of customer requirements. Customer requirements are classified based on the slope of the fitted function curves. In addition, customer requirements can generally be divided into dominant and implicit requirements. Obviously, whether enterprises can meet the implicit requirements becomes an important consideration for improving product quality and retaining customers. Xi et al.^16^ used the triangular fuzzy sets to realize fuzzy semantic quantization of customers and constructed the implicit requirements classification model based on self-organizing mapping neural network. Onyeka et al.^17^ developed a software tool called COTIR that integrates commonsense knowledge, ontology knowledge and text mining for implicit requirements identification. As a matter of fact, customer requirements can be divided into functional, behavioral and structural requirements. Function-Behavior-Structure design process model is a general design solution framework, which assists designers to solve the design task by describing the relationship among product function, behavior and structure^18^. The cognition of designers still follows the mapping process corresponding to the functional domain, behavioral domain and structural domain. Therefore, the customer requirements expression are satisfactory when they are consistent with the cognition of designers. Latent product functional, behavioral and structural requirements are obtained through an analogy-inspired VPA experiment. Those feasible and innovative customer requirements will provide support for designers.

### Deep transfer learning in the natural language processing

With the development of deep learning and high-performance GPU, plentiful neural network models with more layers and more parameters are proposed. However, it is difficult to achieve satisfying result without a large number of data for model training. In terms of this issue, transfer learning method was proposed^19^. Namely, the neural network structure parameters are trained in advance through a large amount of data, and then the trained neural network is fine-tuned under the current specific task. The idea of transfer learning was widely applied in the field of natural language processing when word2vec was displayed^20^. Nevertheless, the word vectors obtained by word2vec are static, which is hard to solve polysemy problem. In response to the polysemy problem, ELMo based on bi-directional long short-term memory structure was presented^21^. Nonetheless, the difficulty of parallel training in ELMo prevents its network depth increasing. Since Transformer network was proposed, the high parallelism of multi-head attention mechanism can learn relevant information in different subspaces and it is designed into a deeper network structure to acquire stronger semantic representation ability^22^. The BERT pre-training language model based on Transformer unit has reached the leading level in many natural language processing tasks due to its excellent semantic representation and transfer generalization ability^23,24^. It is unnecessary for specific tasks to rebuild network structure and basic neural network can be directly designed in the last layer of BERT. Deep transfer learning in the natural language processing is widely utilized in the product design. Wang et al.^25^ explored a method for smart customization service based on configurators. The ELMo was adopted to encode the review text and the mapping between customer requirements and product specifications was built by a multi-task learning-based neural network. Qie et al.^26^ analyzed product textual requirements and created the related models with deep learning and natural language processing skills. In addition, it is worth noting that the dataset is usually imbalanced. On the one hand, granular computing^27–29^ and data resampling^30,31^ are utilized to change the imbalance rate of training dataset. On the other hand, ensemble learning methods can enhance the classification efficacy of imbalanced data by combining a series of weak classifiers^32,33^. Indicators including Precision, Recall and F1 are often applied to evaluate the classifier performance for imbalanced data. In this paper, the text data transformed from VPA data is segmented with natural sentences as the unit and then input into the established BERT deep transfer model. The functional, behavioral and structural customer requirements are classified by fine-tuning the BERT deep transfer model and classifier efficacy for imbalanced text data is evaluated.

### Customer requirements mining

Previously, customer requirements were analyzed by offline ways like questionnaire or interview. In contrast, the voice of customer is contained in a large number of online reviews at present. Sun et al.^4^ proposed a dynamical mining method about ever-changing customer requirements. The changing behavior of product attributes was analyzed and an improvement strategy for next-generation product design was shown based on the changing behavior of attributes. Li et al.^34^ applied general rough set concepts to reveal the association between historical customer needs and design specifications. Jin et al.^35^ identified the product features and sentiment polarities from big consumer requirements data and employed kalman filter method to forecast the consumer requirement trends. As we know from the “Customer requirements classification” section, customer requirements actually involve multi-domain information and functional customer requirements represent customer intention maximally. However, there is a lack of detailed elaboration on the acquisition of functional customer requirements topic-word distribution. Hence, a series of topic models like latent semantic analysis (LSA), probabilistic latent semantic analysis (PLSA) and latent Dirichlet allocation (LDA)^36–38^ can be widely applied to make implicit and fuzzy customer intention explicitly. Topic-word distribution about functional requirements descriptions in the analogy-inspired VPA experiment can be confirmed. Nevertheless, the LSA is not a probabilistic language model so that ultimate results are hard to be explained intuitively. Although the PLSA endows the LSA with probabilistic interpretation, it is prone to overfit due to the solving complexity. Subsequent, the LDA is proposed by introducing Dirichlet distribution into the PLSA. However, traditional LDA is faced with some defects such as the empirical selection of topic quantity, which results in the algorithm performance degradation. To avoid the above problems, this paper presents an ILDA method.

## Method

For this research involving human participants, we identify that this research has been approved by the ethical committee of the Zhejiang University and the state key laboratory of fluid power and mechatronic systems, confirm that this research was performed in accordance with the declaration of Helsinki. Meanwhile, informed consent was obtained from all participants and they agreed to the publication of identifying information/images in an online open-access publication.

### Customer requirements classification based on BERT deep transfer model

With the development of deep learning and high-performance GPU, plentiful neural network models with more layers and more parameters are proposed. However, it is difficult to achieve satisfying result without a large number of data for model training. In fact, the collected customer requirements from the analogy-inspired VPA experiment is a small dataset. In terms of this issue, we adopt deep transfer learning method based on model pre-training. Research on the interpretability of deep neural networks shows that the shallow layers of the networks extract general features, while the deep layers of networks extract high-level semantic information related to specific tasks^39^. Hence, it is not difficult to obtain satisfying results for small dataset under specific tasks by transferring the powerful language representation ability of deep language model to the customer requirements classification. The BERT is an autoencoder language model, which gains semantic representation knowledge by adding noise to the input data and restoring masked information. The Transformer network is the core of the BERT and detailed introduction about the Transformer network can be acquired in the literature21. Compared with the previous mainstream approaches, the Transformer has the following advantages: (1) The limitation of parallel computation of recurrent neural network as well as its derivatives is broken. (2) The number of operations for calculating the association between two positions in the network is not effected by distance. (3) A more explanatory deep learning model is generated through the multi-head self-attention mechanism.

In this paper, there are 12 layers Transformer network in the base-model. The dimension of hidden layer is 768 and there are 12 attention heads in total. Large-model consists of 24 layers Transformer network. The dimension of hidden layer is 768 and there are 16 attention heads in total. The input Chinese sentences are converted into word vectors including token, position and segment, which respectively represent the word itself, word position and sentence dependency. The obtained vector representations are input into the BERT model, and the bi-directional Transformer structure can effectively extract semantic associations in the text data. The Gaussian error linear unit (GELU) is used as a nonlinear activation function inside BERT, which is presented as follows.

1 $$ GELU(x) \approx 0.5x\left( {1 + \tanh \left[ {\sqrt {2{/}\pi } \left( {x + 0.044715x^{3} } \right)} \right]} \right) $$

In the BERT pre-training process, all texts are divided into sentences, in which any two sentences constitute a training data and 15% words are masked randomly in the training data. The pre-training is implemented based on two unsupervised tasks, which are masked language model and next sentence prediction. Multi-source pre-training data makes BERT model more powerful in the semantic representation of Chinese text. In fact, the original Chinese BERT model proposed by Google only uses Chinese Wikipedia as the pre-training corpus. Considering the huge influence of Baidu baike in the Chinese knowledge community, choosing a parallel corpus is more conducive to the domain knowledge transfer. Hence, the BERT pre-training model is carried out on the Chinese Wikipedia and Baidu baike so that the Chinese semantic representation can be fully learned^40^. Moreover, the above two enormous and universal corpus contain abundant textual data related to the functional, behavioral and structural requirements of elevator. Namely, there are sufficient semantic connections between customer requirements and training corpus. In the fine-tuning stage, full connection layers and a softmax layer are added to the output-end of BERT for fine-tuning training. The cross entropy loss function is utilized for back-propagation training and the accuracy is employed to demonstrate the model classification ability. The accuracy and cross entropy loss function are calculated as follows^40^.

2 $$ {\text{Accuracy = }}\frac{1}{m}\sum\limits_{i = 1}^{m} {F\left( {f(x_{i} ) = y_{i} } \right)} $$

3 $$ F\left( {f(x_{i} ) = y_{i} } \right) = \left\{ {\begin{array}{*{20}l} {1,} \hfill & {f(x_{i} ) = y_{i} } \hfill \\ { - 1,} \hfill & {otherwise} \hfill \\ \end{array} } \right. $$

4 $$ CE{ = } - \frac{1}{m}\sum\limits_{i = 1}^{m} {y\log \left( {f(x_{i} )} \right)} $$

**Figure 2:**
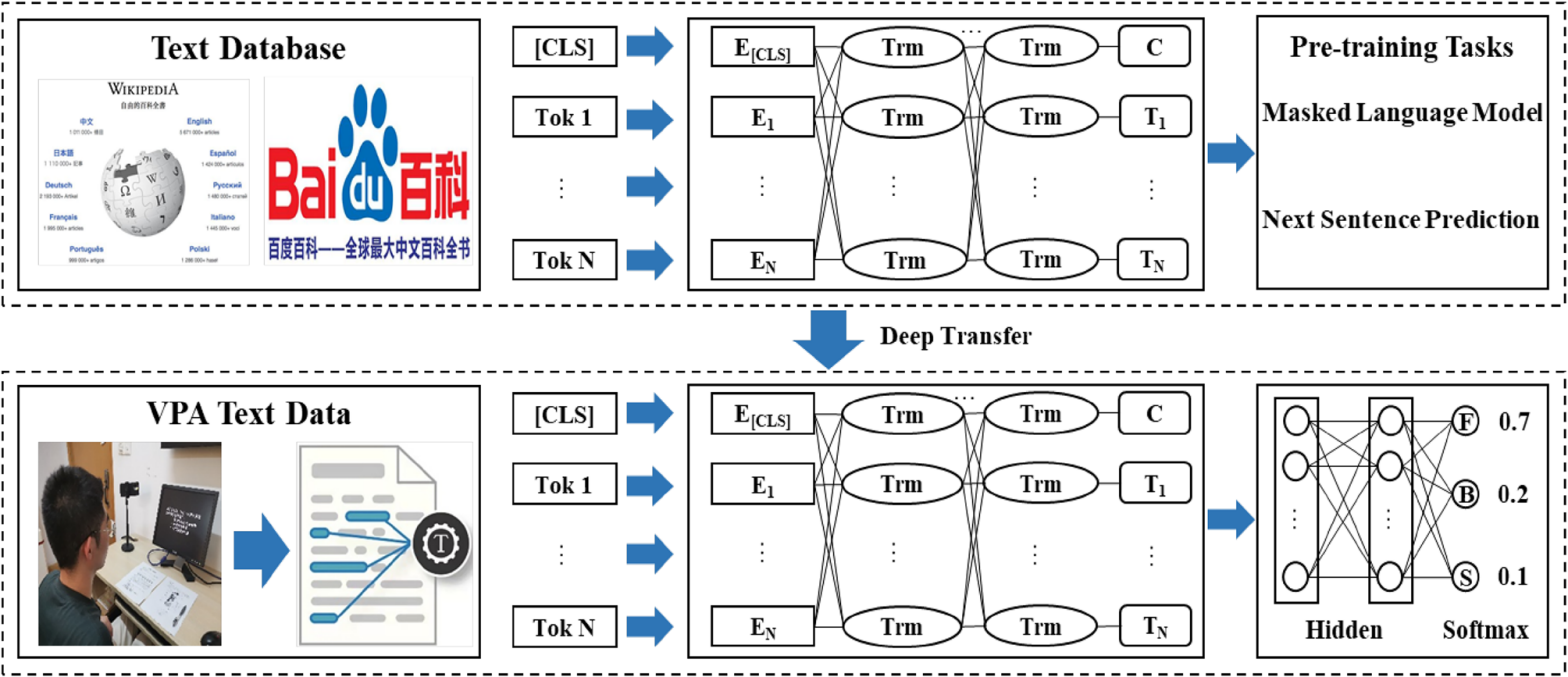
The implementation process of customer requirements classification based on BERT.

The voice of customers is obtained by accomplishing the analogy-inspired VPA experiment and converted into text data. The text data is segmented as many sentences and input into the BERT deep transfer model for fine-tuning, so as to classify customer requirements as functional domain, behavioral domain and structural domain. The implementation process of customer requirements classification based on BERT deep transfer model is shown in Fig. 2.

### Customer requirements mining based on ILDA

Customer requirements mining is actually a topic analysis task aiming at functional customer requirements representing customer intention maximally on the basis of the customer requirements classification. However, traditional LDA is faced with some defects like the empirical selection of topic quantity, which results in the algorithm performance degradation. This paper adopts ILDA method to acquire the topic-keyword distribution representing customer intention. Here are some basic definitions involving the ILDA method primarily^39^. Three sets used in the ILDA are listed as follows. (1) Word set *W* is {*w*_1_, …, *w*_*v*_, …, *w*_*V*_}, *w*_*v*_ represents the *v*th word (*v* = 1, 2, …, *V*) and *V* is the total number of the words. (2) Document set *D* is {**w**_1_, …, **w**_*m*_, …, **w**_*M*_}, **w**_*m*_ indicates the *m*th document (*m* = 1, 2, …, *M*) and *M* is the total number of the documents. Document **w**_*m*_ is regarded as a sequence of words. Namely, **w**_*m*_ is (*w*_*m*1_, …, *w*_*mn*_, …, *w*_*mNm*_), *w*_*mn*_ describes the *n*th word in the document **w**_*m*_ (*n* = 1, 2, …, *N*_*m*_) and *N*_*m*_ is the total number of the words in the document **w**_*m*_. (3)Topic set *Z* is {*z*_1_, …, *z*_*k*_, …, *z*_*K*_}, *z*_*k*_ means the *k*th topic (*k* = 1, 2, …, *K*) and *K* is the total number of the topics. Therefore, the generation process of the document set *D* is expressed as follows. (1) Generate word distributions of *K* topics randomly. Specifically, a vector *φ*_*k*_ (*φ*_*k*_ ~ Dir(*β*)) is randomly produced as the parameter of the word distribution *p*(*w*| *z*_*k*_) of topic *z*_*k*_. (2) Generate topic distributions of *M* documents randomly. Namely, a vector *θ*_*m*_ (*θ*_*m*_ ~ Dir(*α*)) is randomly produced as the parameter of the topic distribution *p*(*z*|**w**_*m*_) of document **w**_*m*_. (3) Generate a sequence of words of *M* documents according the multinomial distributions Mult(*θ*_*m*_) and Mult(*φ*_*k*_). In summary, the joint probability distribution of the model consists of observed variables and latent variables, which is presented in the Eq. (5)^39^.

5 $$ p({\mathbf{w}},{\mathbf{z}}{,}\theta {,}\varphi |\alpha ,\beta ) = \prod\limits_{k = 1}^{K} {p(\varphi_{k} |\beta )} \prod\limits_{m = 1}^{M} {p(\theta_{m} |\alpha )} \prod\limits_{n = 1}^{{N_{m} }} {p(z_{mn} |\theta_{m} )p(w_{mn} |z_{mn} ,\varphi )} $$

where observed variable **w** is a sequence of words of *M* documents, latent variable **z** is a sequence of topics of *M* documents, latent variable *θ* is the parameters of the topic distributions of *M* documents, latent variable *φ* is the parameters of the word distributions of *K* topics, *α* and *β* are the hyper-parameters. Appropriate values for hyper-parameters *α* and *β* depend on the number of topics and the number of words in the corpus. For most applications, good results can be obtained by setting *α* = 50/K + 1 and *β* = 200/V^41^*.*

Hence, the algorithm learning process is to estimate the latent variables **z**, *θ* and* φ* of the joint probability distribution according to the observed variable **w**. In fact, it is a complicated optimization problem and we can only obtain the approximation solutions. Approximate solutions are mainly Gibbs sampling^42^ and variational inference^43^. This paper applies the collapsed Gibbs sampling because of its simple and feasible implementation^42^. The implementation process of the collapsed Gibbs sampling can be briefly described as follows. (1) The marginal probability distribution *p*(**w**, **z**|*α*, *β*) is acquired by integrating the latent variables *θ* and *φ*. (2) The posterior probability distribution *p*(**z**| **w**, *α*, *β*) is sampled to gain the sample set of *p*(**z**| **w**, *α*, *β*). (3) The above sample set is utilized to estimate the latent variables **z**, *θ* and *φ*. The estimation equations for parameter *θ* and *φ* are shown as follows^42^.

6 $$ \theta_{mk} = \frac{{n_{mk} + \alpha_{k} }}{{\sum\nolimits_{k = 1}^{K} {(n_{mk} + \alpha_{k} )} }} $$

7 $$ \varphi_{kv} = \frac{{n_{kv} + \beta_{v} }}{{\sum\nolimits_{v = 1}^{V} {(n_{kv} + \beta_{v} )} }} $$

where *θ*_*mk*_ represents the probability that topic *z*_*k*_ appears in the document **w**_*m*_, *φ*_*kv*_ expresses the probability that word *w*_*v*_ appears in the topic *z*_*k*_, *n*_*mk*_ is the count of the document-topic and *n*_*kv*_ is the count of the topic-word. In addition to the hyper-parameters setting, an essential factor affecting the efficacy of the topic analysis is the optimal topic number *K.* At present, some measurable indicators like Perplexity and KL divergence are adopted to measure the optimal topic quantity^44^. However, Perplexity focuses on the prediction ability of the LDA model for new documents, which often leads to larger topic quantity. Meanwhile, KL divergence pays attention to the difference and stability among topics so that the optimal topic quantity is fewer. Thus, this paper proposes an improved measurable indicator Perplexity-AverKL for gaining the optimal topic quantity by combining the advantages of Perplexity and KL divergence. The detailed calculation equations are displayed as Eqs. (8)–(11).

8 $$ Perplexity = \exp \left( { - \frac{{\sum {\log p(w)} }}{{N_{d} }}} \right) $$

9 $$ AverKL = \frac{{\sum\nolimits_{i = 1}^{K} {\sum\nolimits_{j = 1}^{K} {KL\left( {\left. {z_{i} } \right\|z_{j} } \right)} } }}{{C_{K}^{2} }} $$

10 $$ KL\left( {\left. {z_{i} } \right\|z_{j} } \right) = \sum {p(z_{i} {|}{\mathbf{w}})} \log \frac{{p(z_{i} {|}{\mathbf{w}})}}{{p(z_{j} {|}{\mathbf{w}})}} $$

11 $$ Perplexity - AverKL = \frac{Perplexity}{{AverKL}} $$

where *p*(*w*) is the occurrence probability of each word in the corpus, *N*_*d*_ is the total number of words in the corpus, *z*_*i*_ and *z*_*j*_ is the extracted topics, *KL*(*z*_*i*_‖*z*_*j*_) is the KL divergence between topic *z*_*i*_ and *z*_*j*_, C_*K*_^2^ is the calculation times of *KL* divergence between two topics when the topic quantity is *K*. As can be seen from the above Eqs. (8)–(11), the generalization ability of the ILDA model is stronger when the Perplexity is smaller. Moreover, the difference among topics is greater when the AverKL is bigger. Small average similarity among topics means valid topics extraction. Namely, the optimal topic quantity *K* is determined when Perplexity-AverKL is the smallest.

From what has been discussed above, the specific ILDA implementation procedures are described as follows. (1) Preprocess all documents in the functional requirement corpus by carrying out Chinese words segmentation and deleting the Chinese stop-words. (2) Select the appropriate topic quantity *K* and initialize the hyper-parameters *α* and *β*. (3) Utilize Gibbs sampling to estimate the latent variables **z**, *θ* and *φ*. (4) Change the topic quantity *K*, then repeat step (2) and (3). Different topic analysis results under different topic quantity can be acquired. (5) The optimal topic quantity *K* is determined based on the proposed measurable indicator Perplexity-AverKL. Finally, the first-rank topic analysis result is output. Namely, multiple types of customer intentions and corresponding keywords can be obtained.

## Case study

**Figure 3:**
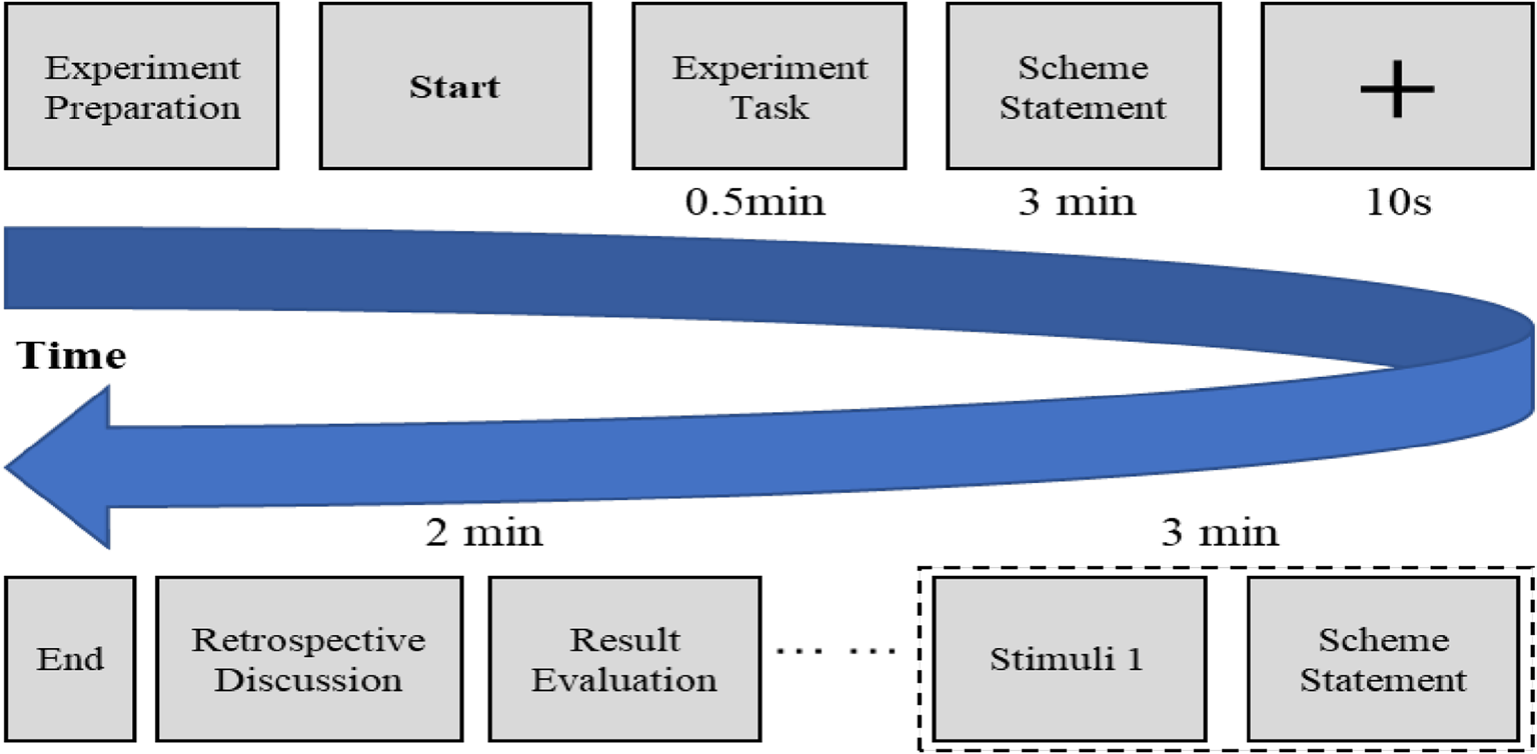
The analogy-inspired VPA experiment process.

In order to conduct the proposed method, this paper implements an analogy-inspired VPA experiment. The experiment goal is to put forward as many functional, behavioral and structural requirements about elevator as possible based on the existing elevator design schemes and analogical inspiration. Ten Chinese graduate students majoring in mechanical engineering are selected as the experiment subjects and numbered from S_1_ to S_10_. They participated in the elevator design project previously and had a deep understanding on the function and structure of elevator. The average age of ten subjects is 24 year-olds and informed consent is obtained from all participants. The experiment is executed in a quiet room so that subjects can think deeply. Experiment instruction and tools like pens and paper are provided to the subjects. The experiment starts after the subjects are fully aware of the experimental specifications, problems and procedures. The spoken data of thinking aloud is collected by video recording during the experiment, and a retrospective discussion is conducted before the end of the experiment so that some errors in the spoken data preprocessing can be avoided. Particularly, the thinking aloud of subjects and experiment materials are all in Chinese in order to reduce the cognitive load of subjects. The analogy-inspired VPA experiment process is displayed in Fig. 3.

**Figure 4:**
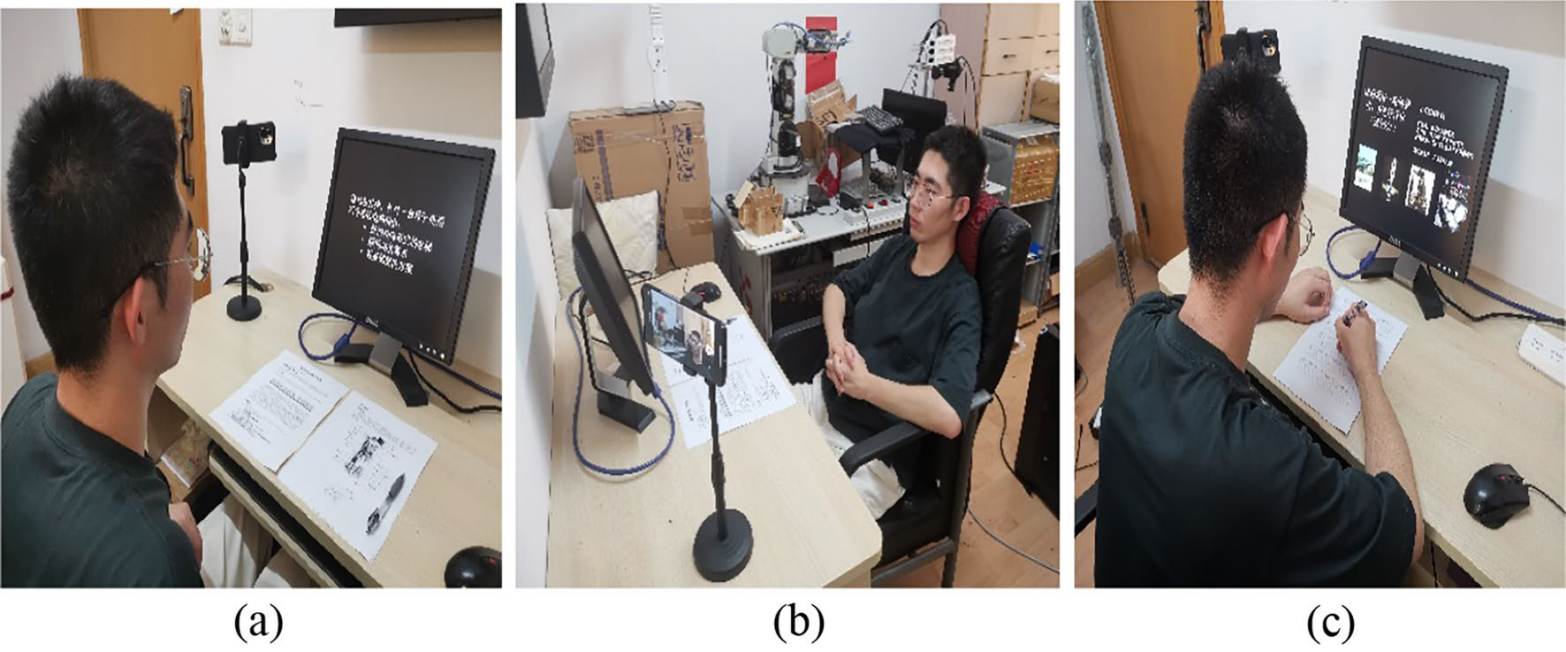
The experimental environment and process: (**a**) experiment preparation, (**b**) requirement statement, (**c**) evaluation and discussion.

The detailed procedures of the experiment are described below: (1) Experiment preparation stage. The experimenter explains the verbal protocol analysis method to the subjects and assists them become familiar with the experiment process. Besides, the subjects are required to read a formal instruction, which introduces an example about how to express elevator customer requirements under an analogical stimulus normatively. The subjects will imitate the expression style of the example during the experiment process so that we can collect more normative VPA data relatively. (2) Open goal stage. The subjects are asked to describe the elevator requirements based on their existing knowledge freely. This stage lasts 3 min and ensures that the subjects have enough time to understand the experimental task and their knowledge memory in the relevant field is awakened. (3) Analogical stimuli stage. There are plentiful common features between near-domain stimuli and design problem, and they all come from the same or similar fields^9^. Product images such as stereo garage, spring shock absorber and vehicle display screen are randomly presented to the subjects as near-domain stimuli. There are few common features between far-domain stimuli and design problem, and they all come from the irrelevant fields^9^. Product images like magnetically levitated train, sneakers with air cushion and automobile airbag are randomly provided to the subjects as far-domain stimuli. Near-domain stimuli are provided to guarantee the feasibility and usefulness of the customer requirements and far-domain stimuli are selected to assure the novelty of the customer requirements. The subjects can associate stimuli source with existing knowledge in the aspects of function, behavior and structure. Meanwhile, the subjects elaborate elevator requirements by thinking aloud. This stage lasts four rounds and spends 3 min each round. (4) Evaluation and discussion stage. The subjects score the proposed elevator requirements in each round and the experimenter reviews the proposed elevator requirements and discusses them with the subjects. The specific experimental environment and process are shown in Fig. 4. (The recognizable face in Fig. 4 is a participant in the study).

The spoken data is converted into text data by using the Web API based on deep full sequence convolutional neural network provided by iFLYTEK open platform^45–47^. The text data is segmented as natural sentences, which are labeled into functional domain, behavioral domain and structural domain according to the specific sentence semantics. Then, Chinese text normalization tool provided by iFLYTEK open platform^45–47^ is utilized to optimize the colloquial text data in order to ensure the classifier performance. Non-fluent factors such as word repetition and semantic redundancy in the colloquial text data are removed, while the linguistic expression style of the colloquial text data is corrected to make it more similar to formal written language. Since the number of the colloquial text data is not large, three experts, who are familiar with elevator conceptual design and possess high-level Chinese writing skill, are invited to check the above text data according to previous instruction example as well as their own understanding ulteriorly. A part of text data is shown in the Table 1. The obtained text data is translated into English due to the Chinese experimental environment.

**Table 1:** A part of text data.

Semantic label	Semantic fragments
Functional requirements	“Intelligent trip will be realized through elevators, and an intelligent building platform can be established, which is associated with the car-hailing software of users”
Behavioral requirements	“Reasonable operation planning of the elevator makes the limited number of elevators meet the operation needs”
Structural requirements	“The hail door needs to ensure a certain gap with the cabin, and then the tensioning wheel should be arranged on the top of the car”

**Figure 5:**
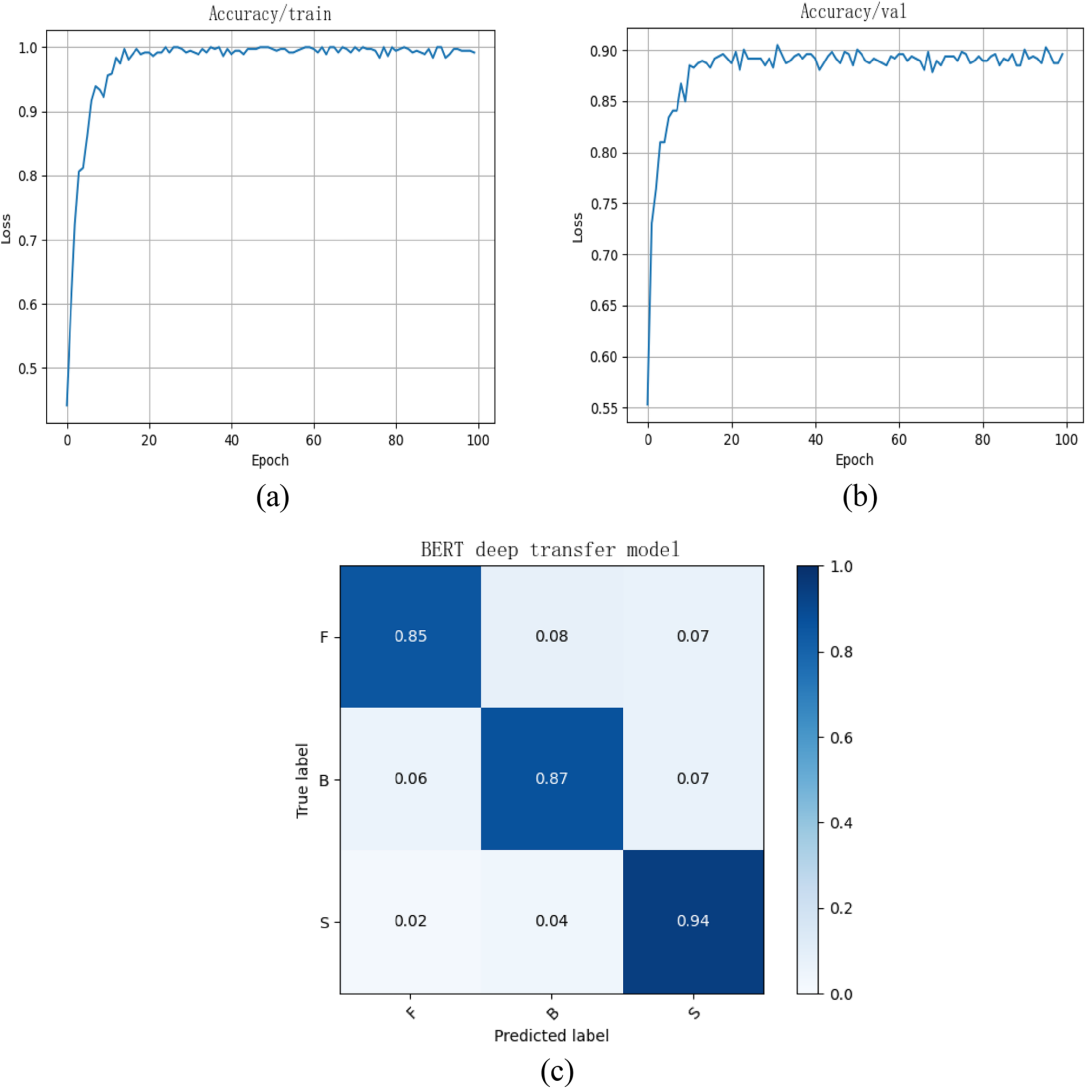
The results of customer requirements classification: (**a**) training accuracy, (**b**) testing accuracy, (**c**) confusion matrix in the testing.

There are a total of 453 labeled sentences, which are randomly divided into training set and test set in a ratio of 8:2. The overall test classification accuracy can reach 88.7% by using the customer requirements classification method proposed in the “Customer requirements classification based on BERT deep transfer model” section. The results of customer requirements classification are presented in Fig. 5. It can be seen from the above results that effective overall classification accuracy has been obtained. Nevertheless, the identification results of functional and behavioral requirements are still not as satisfying as structural requirements, which is toilless to be confused with other types of requirements and lead to misjudgment. The reason for such problem lies in the small-scale semantic dataset currently and the subjective data annotation by some researchers in related fields. Furthermore, it is inevitable that there is potential imbalanced data problem for customer requirements classification. As a matter of fact, the number of functional, behavioral and structural labeled sentences is 103, 161 and 189 respectively. Thus, the evaluation indicators including Precision, Recall and F1 are computed and displayed in the Table 2. The analysis results indicate that the customer requirements classification based on BERT has gained well-pleasing efficacy for potential imbalanced data.

**Table 2:** Precision, Recall and F1 for customer requirements classification.

Label	Precision	Recall	F1
Functional requirements	0.87	0.85	0.86
Behavioral requirements	0.90	0.87	0.88
Structural requirements	0.91	0.94	0.92

**Figure 6:**
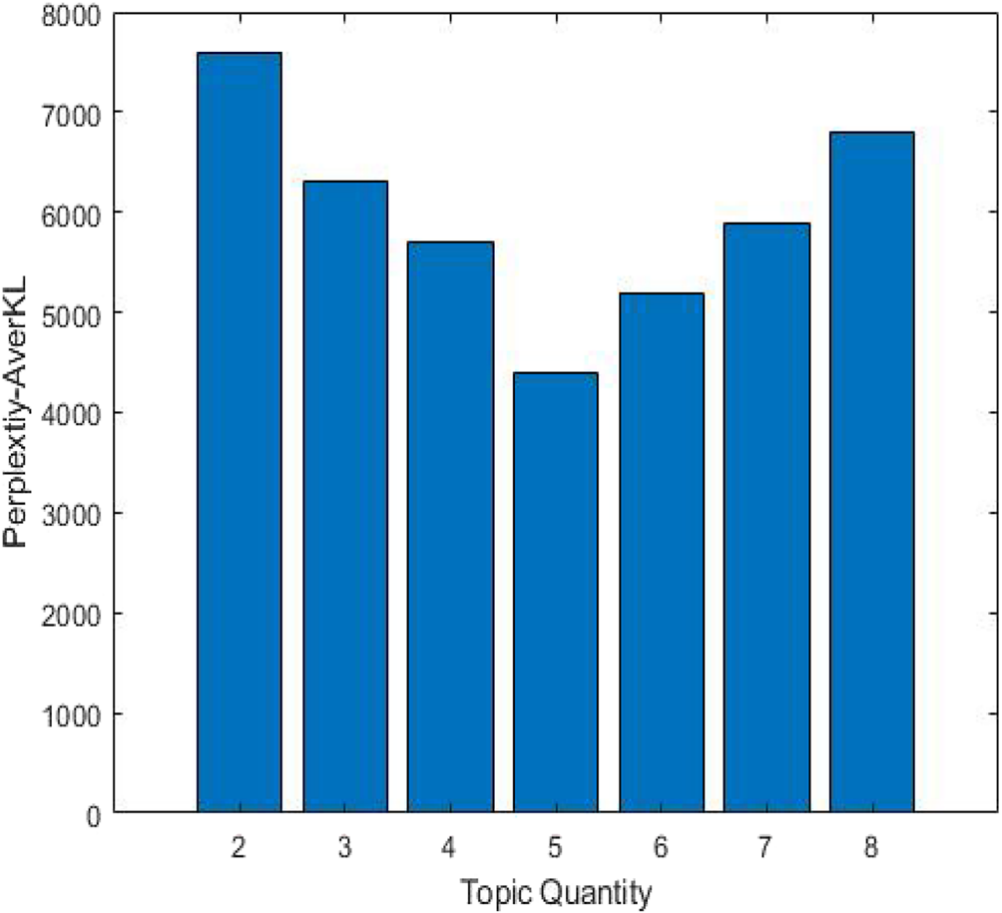
The relationship between the Perplexity-AverKL and the topic quantity.

**Figure 7:**
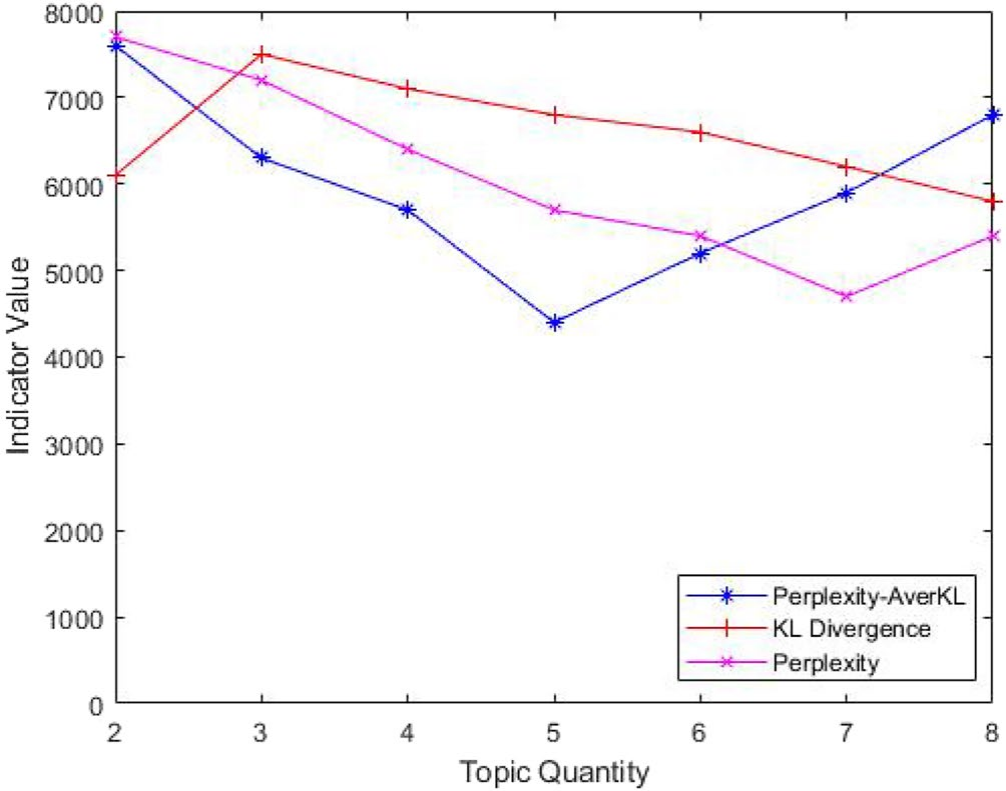
The efficacy comparison among Perplexity-AverKL, Perplexity and KL divergence.

**Figure 8:**
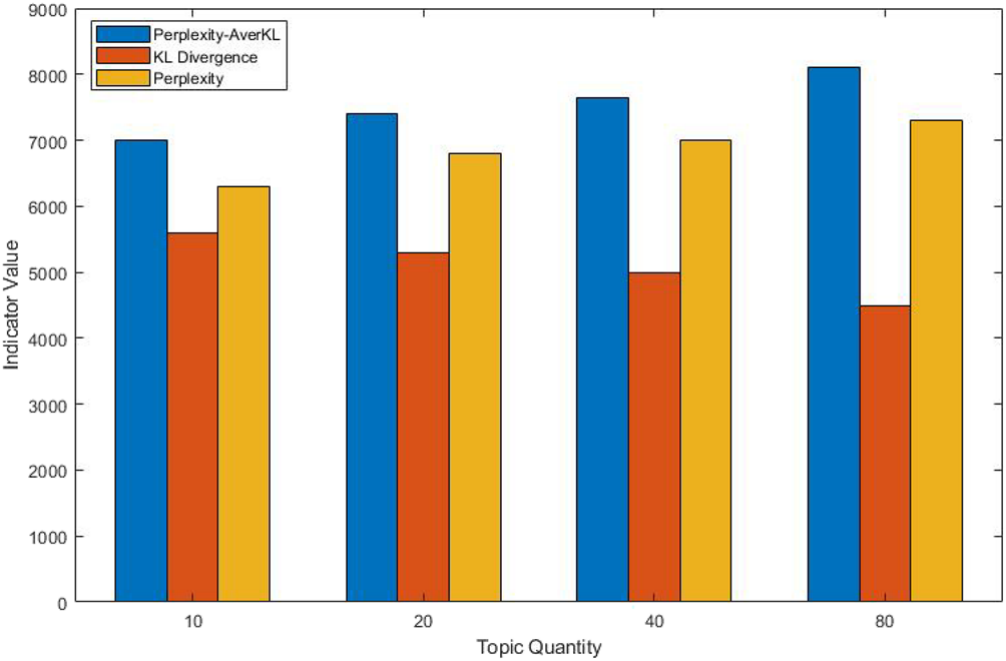
The efficacy comparison among Perplexity-AverKL, Perplexity and KL divergence while setting more topic quantity.

The ILDA method is applied to acquire the functional requirements topic-word distribution representing customer intention maximally. The stop-words method is utilized in order to filter out the words in the functional requirement texts that are not related to the product function. In order to ensure the excellent generalization ability of the ILDA model and the maximal difference among topics, the topic quantity is chosen as five by calculating the Perplexity-AverKL for models with different topic quantity. The relationship between the Perplexity-AverKL and the topic quantity is depicted in Fig. 6. The efficacy comparison among Perplexity-AverKL, Perplexity and KL divergence is presented in Fig. 7. Perplexity focuses on the prediction ability of the LDA model for new documents, which often leads to larger topic quantity. Meanwhile, KL divergence pays attention to the difference and stability among topics so that the optimal topic quantity is fewer. Perplexity-AverKL achieves appropriate topic quantity by combining the advantages of Perplexity and KL divergence. However, although the number of functional requirements texts is not large in this work, different phenomena regarding three different indicators may be presented since the topics from ILDA may mirror the distribution of words rather than the concept about “topic” in our daily conversation. Therefore, it is necessary to further evaluate the performance of the ILDA model with more topic quantity, which is shown in Fig. 8. The results denote that setting more topic quantity does not lead to better model performance due to worse measurable indicator values. We consider why large number of topics are not appropriate from two aspects. On the one hand, the number of types of main functional customer requirements for conceptual design of elevator is not too large. On the other hand, the amount of functional requirements texts is small. Thus, it is not easy to acquire lots of differentiated topics. Finally, five kinds of functional requirements and corresponding keywords as well as their weight coefficients in the topic-word distribution are shown in the Table 3. It can be seen from the table that customer requirements from topic1 to topic 5 are mainly aimed at the elevator operating state, elevator intelligence, elevator internal environment and elevator stability optimization and elevator sightseeing. The requirements analysis results will be beneficial for elevator conceptual design.

**Table 3:** Five kinds of functional requirements.

Topic1	Topic2	Topic3	Topic4	Topic5
Vibration 0.02024	Intelligence 0.02766	Environment 0.02834	Motor 0.02637	Sightseeing 0.02461
Riding 0.01786	Passenger 0.01932	Atmosphere 0.02247	Acceleration 0.02399	Horizon 0.02101
Operation 0.01340	Screen 0.01503	Space 0.01982	Speed 0.02242	Cabin 0.01819
Cabin 0.00987	Device 0.01341	Material 0.01531	Vibration 0.01735	Floor 0.01432
Status 0.00932	Horizon 0.00972	Feeling 0.01194	Steady 0.01325	Experience 0.01014
Device 0.00909	Cabin 0.00945	Experience 0.00939	Filtration 0.00979	Riding 0.00967

## Conclusions

This paper presents a semantic analysis-driven customer requirements mining method for product conceptual design based on deep transfer learning and ILDA. Firstly, an analogy-inspired VPA experiment providing cross-domain stimuli is conducted to obtain feasible and innovative customer requirement descriptions of elevator. Secondly, a BERT deep transfer model is constructed to realize the customer requirements classification among functional domain, behavioral domain and structural domain in terms of the customer requirement descriptions of elevator. Last but not least, the ILDA is proposed to mine the functional customer requirements representing customer intention maximally. Hence, this paper provides a novel research perspective on feasible and innovative customer requirements mining in the product conceptual design through natural language processing algorithm.

There are still some limitations and shortcomings in this work, which should be addressed in the future. On the one hand, the customer requirements acquired from the analogy-inspired VPA experiment are not abundant. More experiments are necessary to be implemented for providing massive and high-quality data. On the other hand, the topic-word distribution of customer requirements is extracted without considering the behavioral and structural customer requirements. The reason is that the semantic presentation in the behavioral and structural domain is usually manifested as “verb-noun” matching form, which is difficult to be extracted directly.

## References

[1] Da-Silva RH, Kaminski PC, Armellini F (2020). Improving new product development innovation effectiveness by using problem solving tools during the conceptual development phase: Integrating design thinking and TRIZ. Creat. Innov. Manag..

[2] Lou SH (2021). An edge-based distributed decision-making method for product design scheme evaluation. IEEE Trans. Ind. Inform..

[3] Lai XJ (2019). The analytics of product-design requirements using dynamics internet data: Application to Chinese smartphone market. Int. J. Prod. Res..

[4] Sun H, Guo W, Shao HY, Rong B (2020). Dynamical mining of ever-changing user requirements: A product design and improvement perspective. Adv. Eng. Inform..

[5] Zhou F, Jiao RJ, Linsey JS (2015). Latent customer needs elicitation by use case analogical reasoning from sentiment analysis of online product reviews. J. Mech. Des..

[6] Christensen BT, Ball LJ (2016). Creative analogy use in a heterogeneous design team: The pervasive role of background domain knowledge. Des. Stud..

[7] Cash P, Kreye M (2018). Exploring uncertainty perception as a driver of design activity. Des. Stud..

[8] Yuan P, Li Y, Chen J, Xiong Y, Liu LF (2018). Experimental study on the associations among sketches based on design cognition. J. Mech. Des..

[9] Goucher-Lambert K, Moss J, Cagan J (2019). A neuroimaging investigation of design ideation with and without inspirational stimuli-understanding the meaning of near and far stimuli. Des. Stud..

[10] Chai CL, Cen F, Ruan WY, Yang C, Li HT (2015). Behavioral analysis of analogical reasoning in design: Differences among designers with different expertise levels. Des. Stud..

[11] Sanderson D, Chaplin JC, Ratchev S (2019). A function-behavior-structure design methodology for adaptive production systems. Int. J. Adv. Manuf. Technol..

[12] Violante MG, Vezzetti E (2017). Kano qualitative vs quantitative approaches: An assessment framework for products attributes analysis. Comput. Ind..

[13] Xu, Q. L. *et al*. Customer requirements analysis based on an analytical Kano model. In *Proceedings of the IEEE International Conference on Industrial Engineering and Engineering Management*, vol. 2–5-December-2007 (2007).

[14] Lou SH, Feng YX, Zheng H, Gao YC, Tan JR (2020). Data-driven customer requirements discernment in the product lifecycle management via intuitionistic fuzzy sets and electroencephalogram. J. Intell. Manuf..

[15] Shi YL, Peng QJ (2021). Enhanced customer requirements classification for product design using big data and improved Kano model. Adv. Eng. Inform..

[16] Xi, T. & Wang, L. J. The classification model of customer groups based on grader. In *Proceedings of the 2th International Conference on Artificial Intelligence and Industrial Engineering*, vol. 133 (2016).

[17] Onyeka, E., Varde, A. S., Anu, V., Tandon, N. & Daramola, O. Using commonsense knowledge and text mining for implicit requirements localization. In *Proceedings of the IEEE International Conference on Tools with Artificial Intelligence*, vol. 9–11-November-2020 (2020).

[18] Sanderson D, Chaplin JC, Ratchev S (2019). A function-behaviour-structure design methodology for adaptive production systems. Int. J. Adv. Manuf. Technol..

[19] Pan SJ, Yang Q (2010). A survey on transfer learning. IEEE Trans. Knowl. Data Eng..

[20] Mikolov, T., Chen, K., Corrado, G. & Dean, J. *Efficient Estimation of Word Representations in Vector Space*. Preprint at https://arXiv.org/abs/1301.03781 (2013).

[21] Peters, M. E. *et al*. *Deep Contextualized Word Representations*. Preprint at https://arXiv.org/abs/1802.05365 (2018).

[22] Vaswani, A. *et al*. *Attention is All You Nee*d. Preprint at https://arXiv.org/abs/1706.03762 (2017).

[23] Devlin, J., Chang, M. W., Lee, K. & Toutanova, K. *BERT: Pre-training of Deep Bidirectional Transformers for Language Understanding*. Preprint at https://arXiv.org/abs/1810.04805 (2018).

[24] Cui, Y. M. *et al*. *Pre-training with Whole Word Masking for Chinese BERT*. Preprint at https://arXiv.org/abs/1906.08101 (2019).

[25] Wang Y, Li X, Tsung F (2020). Configuration-based smart customization service: A multitask learning approach. IEEE Trans. Autom. Sci. Eng..

[26] Qie, Y. J. *et al*. A deep learning based framework for textual requirements analysis and model generation. In *Proceedings of the IEEE CSAA Guidance, Navigation and Control Conference*, vol. 10–12-August-2018 (2018).

[27] Leng JW, Chen QX, Mao N, Jiang PY (2018). Combining granular computing technique with deep learning for service planning under social manufacturing contexts. Knowl. Based Syst..

[28] Leng JW, Jiang PY (2017). Granular computing-based development of service process reference models in social manufacturing contexts. Concurr. Eng. Res. Appl..

[29] Leng JW (2021). A loosely-coupled deep reinforcement approach for order acceptance decision of mass-individualized printed circuit board manufacturing in industry 4.0. J. Clean. Prod..

[30] Boughorbel S, Jarray F, El-Anbari M (2017). Optimal classifier for imbalanced data using Matthews Correlation Coefficient metric. PLoS ONE.

[31] Lin WC, Tsai CF, Hu YH, Jhang JS (2017). Clustering-based undersampling in class-imbalanced data. Inf. Sci..

[32] Sun J, Li H, Fujita H, Fu BB, Ai WG (2019). Class-imbalanced dynamic financial distress prediction based on Adaboost-SVM ensemble combined with SMOTE and time weighting. Inf. Fusion.

[33] Dong XB, Yu ZW, Cao WM, Shi YF, Ma QL (2020). A survey on ensemble learning. Front. Comput. Sci..

[34] Li JR, Wang QH (2010). A rough set based data mining approach for house of quality analysis. Int. J. Prod. Res..

[35] Jin J, Liu Y, Ji P, Lin HG (2016). Understanding big consumer opinion data for market-driven product design. Int. J. Prod. Res..

[36] Landauer TK, Foltz PW, Laham D (1998). An introduction to latent semantic analysis. Discl. Process..

[37] Hofmann T (2001). Unsupervised learning by probabilistic latent semantic analysis. Mach. Learn..

[38] Blei DM, Ng A, Jordan MI (2003). Latent Dirichlet allocation. J. Mach. Learn. Res..

[39] Samek W, Montavon G, Lapuschkin S, Anders CJ, Muller KR (2021). Explaining deep neural networks and beyond: A review of methods and applications. Proc. IEEE.

[40] Zhang, Z. Y. *et al*. ERNIE: Enhanced language representation with informative entities. In *Proceedings of the 57th Annual Meeting of the Association for Computational Linguistics*, vol. 28–31-July-2019 (2019).

[41] Asuncion, A., Welling, M., Smyth, P. & Teh, Y. W. On smoothing and inference for topic models. In *Proceedings of the 25th Conference on Uncertainty in Artificial Intelligence*, vol. 18–21-June-2009 (2009).

[42] Porteous, I. *et al*. Fast collapsed gibbs sampling for latent Dirichlet allocation. In *Proceedings of the 14th ACM SIGKDD International Conference on Knowledge*, vol. 24–26-August-2008 (2008).

[43] Blei DM, Kucukelbir A, McAuliffe JD (2017). Variational inference: A review for statisticians. J. Am. Stat. Assoc..

[44] Jelodar H (2019). Latent Dirichlet allocation (LDA) and topic modeling: Models, applications, a survey. Multimed. Tools Appl..

[45] Pan, W. J., Tan, J. W., Zhang, Q. Y. & Luan, T. Research of a mobile ATC communication training system. In *International Conference on Advanced Manufacturing Technology and Industrial Application*, vol. 25–26-September-2016 (2016).

[46] Wang, L. & Luo, H. R. Design of intelligent vehicle for distribution system based on speech recognition. In *Proceedings of 2017 IEEE International Conference on Computer and Communications*, vol. 13–16-December-2017 (2017).

[47] Zhao, T. *et al*. A design and implementation of intelligent cradle. In *Proceedings of 2020 International Conference on Artificial Life and Robotics*, vol. 13–16-January-2020 (2020).

